# Predicting Miscibility of Ionic Liquids in Binary Mixtures

**DOI:** 10.1021/acs.jcim.6c00050

**Published:** 2026-04-14

**Authors:** Ahaduzzaman Nahid, Ashfaq Iftakher, Puneeth Ganesh Babu Agoram, Luke W. Wallisch, Abby N. Harders, Kalin R. Baca, M. M. Faruque Hasan

**Affiliations:** † Artie McFerrin Department of Chemical Engineering, 14736 Texas A&M University, College Station, Texas 77843-3122, United States; ‡ 4202 Icorium Engineering Company, Lawrence, Kansas 66047, United States; § Texas A&M Energy Institute, Texas A&M University, College Station, Texas 77843, United States

## Abstract

Blending two ionic liquids (ILs) offers a promising route to discover superior solvents by combining complementary molecular features of each IL to achieve tailored thermophysical properties of mixed solvents. However, an exhaustive exploration of the vast combinatorial and compositional design space of IL–IL pairs through experiments or high-fidelity simulations is prohibitively time-consuming. We propose a quantum chemical and thermodynamic data-driven machine learning (ML) framework for predicting the miscibility of binary IL mixtures based on activity coefficients. Specifically, we train an artificial neural network (ANN) using COSMO-RS-generated mixture activity coefficients for over 4.9 million IL–IL compositions. The approach is further generalized across temperatures by using excess enthalpy. The trained ANN shows high accuracy as a threshold-based binary miscibility classifier. We further explore the similarity across IL mixtures to understand how molecular descriptors and mixture compositions correlate with miscibility. By combining ML-based structural analysis, we go beyond the 150 years old solubility principle of *like dissolves like* and deconvolute the roles of molecular identity in determining the miscibility of ILs. Finally, we perform miscibility experiments for several important binary IL–IL mixtures. The experimental observations are largely consistent with the miscibility trends predicted by the computational model.

## Graphical abstract

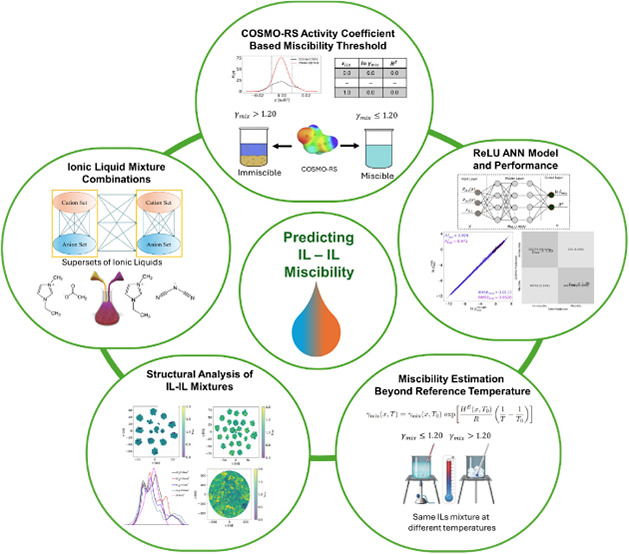

## 1 Introduction

Ionic Liquids (ILs) are salts with melting points typically below 100 °C.^1^ ILs have shown promise as solvents for separating azeotropic mixtures.^2–4^ They have gained significant attention from both industry and academia due to their low vapor pressure, high absorption capacity, high selectivity, and wide structural tunability.^5^

However, pure ILs often lack the optimal balance of selectivity, viscosity, and mass transfer efficiency required for complex separations.^6^ By combining different ILs as mixed solvents, it is possible to exploit complementary properties and fine-tune solvent performance in ways a single IL cannot achieve.^7^ For example, one IL may have high absorption selectivity but slow transport due to high viscosity, while another may have fast mass transfer due to low viscosity but exhibit low selectivity.^8^ By combining them, it is possible to achieve superior separation performance overall due to better handling of the process-scale trade-offs. For example, Larriba et al.^9^ studied the binary IL mixture of 1-ethyl-4-methylpyridinium bis­(trifluoromethylsulfonyl)­imide ([4-Mepy]­[Tf_2_N]) and 1-ethyl-3-methylimidazolium dicyanamide ([C_2_mim]­[DCA]) for the extraction of aromatic hydrocarbons from pyrolysis gasoline. Their results demonstrated that using 75 mol % of [4-Mepy]­[Tf_2_N] and 25 mol % of [C_2_mim]­[DCA] improved the recovery and purity of aromatics compared to using a single IL. In their pioneering work of modeling different properties of IL–IL binary mixtures, Chen et al.^10^ identified a binary solvent comprising butylethylammonium tetrafluoroborate ([N_4,1,0,0_]­[BF_4_]) and 1-methylimidazolium bis­(trifluoromethanesulfonyl)­imide ([C_1_mim]­[Tf_2_N]) that offered improved separation performance for H_2_ recovery from raw coke oven gas. Lee et al.^11^ investigated refrigerant absorption and regeneration, demonstrating substantially improved performance when using ionanofluid–ionic liquid (IONF–IL) mixtures as solvents. Several mixtures of ILs also reduce solvent consumption and remain in the liquid phase across broad composition and temperature ranges.^12^ However, some mixtures become immiscible at specific compositions and temperatures,^13^ and lead to two phases, thereby reducing the overall efficiency of separation processes.^14^ Therefore, it is necessary to determine the miscibility of ILs across temperatures and compositions to assess the overall feasibility of IL-based mixed solvents.

For over 150 years, the design of mixed solvents has been guided by a simple solubility rule: *Like dissolves like*. While this works largely for volatile organic solvents, we lack a mechanistic explanation for the basis that results from the ionic constituents (cations and anions) of numerous candidate ILs to be used under different process conditions. In fact, research on IL–IL mixtures remains comparatively limited and scattered.^15^ Aparicio and Atilhan combined experiments and simulations to probe the molecular structuring of binary IL mixtures and the role of cation/anion architecture,^16^ but their analysis focused primarily on pyridinium-based cations. Omar et al. performed a systematic COSMO-RS/CA study of IL–IL mixing and inferred miscibility from excess molar enthalpies.^17^ However, the CA method is computationally demanding and poses a bottleneck for high-throughput screening. Klamt and co-workers demonstrated that surface charge distributions and σ-profiles effectively capture intermolecular interactions and solubility behavior.^18–20^ Reichardt and Welton applied the like-dissolves-like concept to rationalize solvation phenomena in ILs.^21^ Koddermann et al. used COSMO-RS σ-profiles to rationalize IL solubility trends.^22^ For IL–IL systems, prior works mostly reported thermophysical properties (surface tension, viscosity, density)^23–25^ and gas solubilities (e.g., CO_2_).^26–28^ To the best of our knowledge, there remains a need for computationally efficient methods to predict IL–IL miscibility across compositions and temperatures.

Miscibility is a mixture property that is governed by the excess Gibbs free energy (*G* ^*E*^). A positive deviation of *G* ^*E*^ from zero indicates a tendency toward immiscibility.^29^ Since *G* ^*E*^ depends on the activity coefficients of the species present in the mixture, activity coefficients provide an effective approach to predict miscibility. As the experimental data on activity coefficients are limited,^30^ COSMO-RS^31^ may be utilized to provide a tractable way to compute these for a wide range of IL mixtures.^32^

In this work, we first evaluate the COSMO-RS predictions against experimental data and develop predictive models for binary IL mixture miscibility. In particular, we validate the COSMO-RS-based predictions using experimentally determined miscibility for binary IL–IL mixtures across compositions at 298.15 K. We also generate a large synthetic data set of COSMO-RS-based activity coefficients for different IL–IL mixtures by combining a wide range of cations and anions. We train an artificial neural network (ANN) model to predict IL–IL mixture activity coefficients, which are then used to train a binary miscibility classifier for miscibility prediction. Because miscibility depends on temperature, we extend predictions away from 298.15 K using a van’t Hoff-type relation derived from the Gibbs–Helmholtz equation.^33^ The temperature correction assumes a small Δ*T* and an approximately constant excess molar enthalpy *H* ^*E*^. We validate this correction against direct COSMO-RS calculations at multiple temperatures. We also examine structural signatures of miscibility by comparing σ-profiles and visualizing them with dimensionality reduction techniques such as t-SNE. Overall, our approach enables fast screening of IL–IL miscibility with substantially reduced computational cost.

The remainder of the paper is organized as follows. Section 2 describes the theoretical underpinnings of activity-coefficient-based miscibility classification as well as the validity of the COSMO-RS-based data curation of activity coefficients for IL–IL mixtures. Section 3 details the methodology for predicting IL–IL miscibility in binary mixtures. The methodology includes the ANN model development, temperature translation derivation, and dimensionality reduction techniques. Section 4 presents the results and discusses the model performance, experimental validation, and analytics, as well as the temperature translation correlation outputs. The major conclusions of the paper are summarized in Section 5.

## 2 Theory

The miscibility of IL mixtures can be quantitatively evaluated through the activity coefficient, which measures deviations from ideal solution behavior.^34^ In liquid–liquid phase equilibrium, the thermodynamic stability of a mixture is governed by the excess Gibbs free energy, *G* ^*E*^.^35,36^ Thermodynamically, *G* ^*E*^ can be defined with the activity coefficient and mole fraction of the mixture as follows:

1 $$\frac{G^{E}}{RT}=\underset{i}{\sum }x_{i}\;\ln\gamma_{i}=\ln\gamma_{mix}$$

where *x*_*i*_ is the mole fraction of component *i*, γ_*i*_ is the activity coefficient of component *i*, and γ_*mix*_ is the overall mixture activity coefficient.

When the summation ∑_*i*_ *x*_*i*_ ln γ_*i*_ is positive, the excess Gibbs free energy *G* ^*E*^/*RT* is also positive, indicating a thermodynamic driving force toward immiscibility. This assumed monotonic relationship between the mixture activity coefficient and miscibility is based on the general premise that increasing nonideality reflects increasingly unfavorable intermolecular interactions and, therefore, reduced miscibility. However, we acknowledge that this is a simplified, first-order assumption and that deviations may occur in more complex IL–IL systems.

The activity coefficient is a dimensionless parameter that quantifies molecular interactions within a mixture.^37^ For an ideal mixture, all components behave uniformly as a single homogeneous phase (γ_*i*_ = 1), resulting in an excess Gibbs energy of zero (*G* ^*E*^ = 0). In real systems, however, intermolecular interactions deviate from ideality. When γ_*i*_ < 1, interactions between unlike species are favorable, thereby decreasing *G* ^*E*^ and enhancing miscibility. Conversely, when γ_*i*_ > 1, weaker interactions between unlike species increase *G* ^*E*^ and promote liquid–liquid phase separation.

For an IL mixture at temperature *T* and with each IL composition *x*_*i*_, the activity coefficient follows from the excess chemical potentials as in eq 2:

2 $$\ln\gamma_{i}\left(x_{i},T\right)=\frac{\mu_{i}^{\mathrm{ex}}\left(x_{i},T\right)-\mu_{i}^{\mathrm{ex},\mathrm{pure}}\left(T\right)}{RT}$$

where $\mu_{i}^{\mathrm{ex},\mathrm{pure}}\left(T\right)$ is the excess chemical potential of pure IL_*i*_ at temperature *T*. This formulation yields the activity coefficient (γ_*i*_) of each component IL_*i*_ at finite composition *x*_*i*_ in the mixture.

The excess contribution $\mu_{i}^{\mathrm{ex}}\left(x_{i},T\right)$ is evaluated by integrating pairwise surface contacts over the σ-profiles^31^ as follows:

3 $$\mu_{i}^{\mathrm{ex}}\left(x_{i},T\right)=\iint \left(E_{\mathrm{misfit}}\left(\sigma ,\sigma^{\prime }\right)+E_{\mathrm{HB}}\left(\sigma ,\sigma^{\prime }\right)+E_{\mathrm{vdW}}\left(\sigma ,\sigma^{\prime }\right)\right)p_{i}\left(\sigma \right)p_{\mathrm{mix}}\left(\sigma^{\prime };x_{i}\right)d\sigma d\sigma^{\prime }+\mu_{i,\mathrm{comb}}^{\mathrm{ex}}\left(x_{i},T\right)$$

where *E*_misfit_, *E*_HB_, and *E*_vdW_ denote electrostatic misfit, hydrogen-bonding, and dispersion (van der Waals) interactions, respectively, and $\mu_{i,\mathrm{comb}}^{\mathrm{ex}}$ is the combinatorial (size/shape) term. For a liquid mixture with mole fractions $\{x_{i}{\}}_{i=1}^{N}$ , the composition-dependent σ-profile is the surface-area-weighted average of the component profiles,

4 $$p_{\mathrm{mix}}\left(\sigma \right)=\frac{\overset{N}{\underset{i=1}{\sum }}x_{i}A_{i}{\mathrm{p̃}}_{i}\left(\sigma \right)}{\overset{N}{\underset{i=1}{\sum }}x_{i}A_{i}}=\overset{N}{\underset{i=1}{\sum }}w_{i}{\mathrm{p̃}}_{i}\left(\sigma \right),,w_{i}=\frac{x_{i}A_{i}}{\overset{N}{\underset{j=1}{\sum }}x_{j}A_{j}}$$

where *A*_*i*_ is the total COSMO surface area of component *i* and *p̃*_*i*_(σ) is its unit-area–normalized profile (∫ *p̃*_*i*_(σ) *d*σ = 1). In this work, we use unit-normalized profiles as exported by COSMO-RS, so eq 4 is evaluated directly as *p*_mix_(σ) = ∑_*i*_ *w*_*i*_ *p*_*i*_(σ). Eqs 3 and 4 link molecular σ-profiles to mixture activity coefficients in a composition-consistent manner suitable for assessing IL miscibility.

Conceptually, γ_mix_ ≤ 1.0 should indicate the miscibility for ideal mixtures. However, for real mixtures, this threshold may deviate due to nonideality. Based on a comprehensive survey of 77 experimentally reported miscible and immiscible IL–IL systems, we introduce a practical threshold for the mixture activity coefficient, γ_mix_. Mixtures with γ_mix_ ≤ 1.20 are considered miscible, whereas those with higher values are classified as immiscible. This threshold effectively links the thermodynamic criterion to experimentally observed phase behavior. For each of the 77 experimentally obtained mixtures, COSMO-RS is used to calculate the activity coefficients of both components at various compositions, totaling 1891 data points (see Figure 1). The overall mixture activity coefficient (γ_mix_) is then determined by using the individual activity coefficients of the IL components and their compositions, as shown in eq 1.

**Figure 1:**
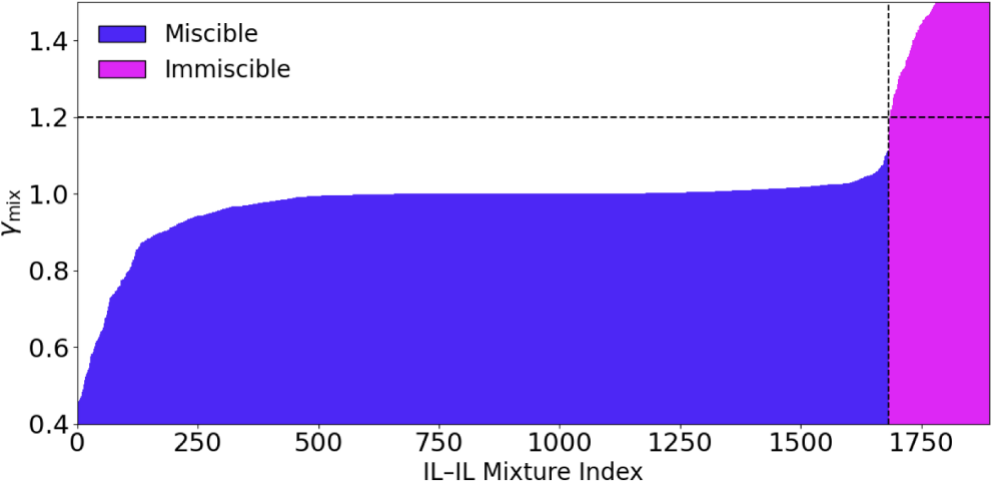
Distribution of activity coefficients (γ_mix_) for experimentally validated IL–IL mixtures at different compositions. All experimentally validated IL–IL mixtures are arranged in ascending order of γ_mix_. The red dashed line represents the miscibility threshold (γ_mix_ = 1.20), separating the miscible (left, blue) and immiscible (right, purple) systems. Miscible mixtures exhibit γ_mix_ ≤ 1.20, whereas immiscible mixtures show distinctly higher activity coefficients.

In this context, small values of γ_mix_ correspond to favorable interactions and single-phase behavior, whereas large values indicate a greater tendency toward phase separation. Based on both experimental evidence and COSMO-RS predictions, mixtures are classified as miscible when γ_mix_ ≤ 1.20 and immiscible when γ_mix_ > 1.20 at a given composition. This physically meaningful threshold provides a clear connection between the computed thermodynamic properties and observed phase behavior.

The γ_mix_ threshold is empirical in nature, which helps define a practical, first-order classification criterion for miscibility. Its value is obtained based on the set of experimentally reported data currently available. We also acknowledge the imbalance between miscible and immiscible data points. To that end, the threshold is more of a guideline rather than a universal boundary and may be refined as additional data become available.

Temperature influences molecular interactions and, consequently, the magnitude of the activity coefficients.^38,39^ To be able to extend the prediction beyond a reference temperature (298.15 K), we derive the following thermodynamic approach for temperature correction. This is based on an analogous form of the van’t Hoff equation^40^ that is typically used to calculate equilibrium constants as a function of temperature. By analogy, the temperature dependence of activity coefficients at fixed composition and pressure follows from the Gibbs–Helmholtz equation applied to excess functions:

$$\frac{G^{E}\left(x,T\right)}{RT}=\underset{i}{\sum }x_{i}\;\ln\gamma_{i}\left(x_{i},T\right)\equiv \ln\gamma_{\mathrm{mix}}\left(x,T\right)$$ 5

6 $$H^{E}\left(x,T\right)=-RT^{2}\frac{\partial }{\partial T}\left(\frac{G^{E}\left(x,T\right)}{RT}\right)$$

Defining ln γ_mix_(*x*,*T*) = ∑_*i*_ *x_i_* ln γ_*i*_(*x_i_*,*T*) and assuming *H* ^*E*^ is approximately constant between *T*_0_ and *T* lead to

7 $$\ln\gamma_{\mathrm{mix}}\left(x,T\right)=\ln\gamma_{\mathrm{mix}}\left(x,T_{0}\right)+\frac{H^{E}\left(x,T_{0}\right)}{R}\left(\frac{1}{T}-\frac{1}{T_{0}}\right)$$

Equivalently, we get

8 $$\gamma_{\mathrm{mix}}\left(x,T\right)=\gamma_{\mathrm{mix}}\left(x,T_{0}\right)\exp\left[\frac{H^{E}\left(x,T_{0}\right)}{R}\left(\frac{1}{T}-\frac{1}{T_{0}}\right)\right]$$

Eq 8 is used to compute miscibility from the activity coefficient beyond the reference temperature.

## 3 Methods

Figure 2 presents the overall workflow of the IL–IL miscibility prediction framework. ILs are composed of cations and anions. Therefore, binary IL–IL mixtures consist of two cations and two anions at varying ratios. Starting with a large pool of cations and anions, we first generate an extensive data set consisting of 4.9 million IL mixtures by systematically combining different concentrations of cations and anions (Figure 2A). Experimentally validated IL–IL mixtures are also identified to define a miscibility benchmark derived from activity coefficients. For each mixture, COSMO-RS is employed to compute the activity coefficients at different compositions (Figure 2B). The resulting mixture activity coefficients (γ_mix_) are analyzed to distinguish between miscible and immiscible systems, where γ_mix_ ≤ 1.20 indicates miscibility. Each IL in the mixture is represented by a 50-bin, unit-normalized σ-profile that captures the surface charge distribution. The σ-profile and mole fraction of the mixture form the input features of a ReLU-based ANN implemented in **PyTorch**. The ANN model is trained to predict both γ_mix_ and excess molar enthalpy (*H* ^*E*^). Model validation is performed by using parity plots and confusion matrices to ensure accurate regression and reliable binary classification of miscibility (Figure 2C). Structural similarity analyses, such as t-SNE visualization, are then applied to elucidate how molecular features influence miscibility behavior across different IL combinations (Figure 2D). Finally, a van’t Hoff-type correlation is formulated to estimate temperature-dependent activity coefficients beyond the reference temperature of 298.15 K (Figure 2E).

**Figure 2:**
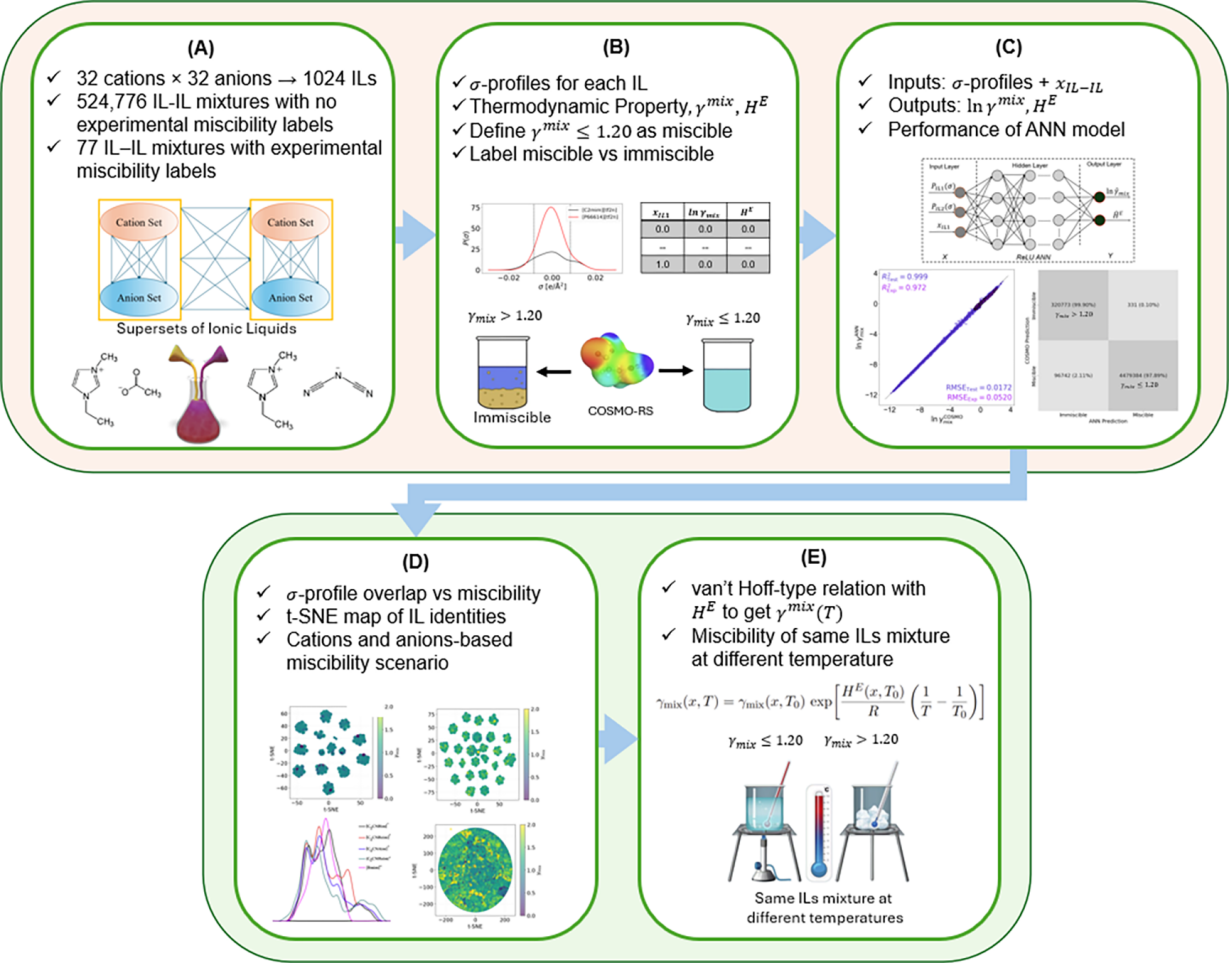
Workflow for the data-driven modeling and prediction of the miscibility of ILs in binary mixtures at different compositions and temperatures. (A) Ionic liquid mixture combinations. (B) COSMO-RS activity coefficient based miscibility threshold. (C) ReLU ANN regression machine learning model. (D) Structural analysis of IL­­–IL mixtures. (E) Miscibility estimation beyond reference temperature.

This integrated thermodynamic–machine learning framework establishes a direct link between activity coefficients and the miscibility of binary IL mixtures. We detail the miscibility criterion, data set and descriptor construction, ANN learning architecture and training protocol, binary miscibility classifier modeling, temperature correction, and evaluation and visualization procedures in the subsequent subsections.

### 3.1 Data Set Preparation

We consider 32 familiar cations and 32 familiar anions (lists are provided in Table S1 and Supporting Information) to compute activity coefficients using COSMO-RS. These cations and anions produce 1024 unique ILs and 524,776 binary IL–IL combinations. From these 524,776 combinations, 170,000 combinations are randomly picked and their activity coefficients are computed at 29 different molar compositions, totaling 4.9 million data points. The TZVP fine parametrization^41^ is used, and the temperature is fixed at 298.15 K.

#### 3.1.1 Miscibility Data Curation

For evaluating the suitability of COSMO-RS in predicting the mutual miscibility of IL–IL pairs, a data set comprising experimental measurements for 77 binary IL–IL systems with markedly different mixing behaviors is compiled through an extensive literature survey. Most of the investigated systems are reported to be completely miscible or immiscible for equimolar mixture compositions at approximately room temperature. A total of 28 cations from the imidazolium, pyridinium, pyrrolidinium, phosphonium, and ammonium families, together with 24 different anions, are combined to form 64 distinct ILs and 77 binary mixtures. The full list of cations, anions, and their abbreviations is provided in Table S2 of the Supporting Information. Among these mixtures, 61 are experimentally identified as miscible, while 16 are found to be immiscible. The activity coefficients of all systems are subsequently calculated using COSMO-RS to assess its ability to predict miscibility and immiscibility. The experimentally validated systems are further categorized into three groups: mixtures with common cations ([A]­[X] + [A]­[Y]), mixtures with common anions ([A]­[X] + [B]­[X]), and mixtures with both distinct cations and anions ([A]­[X] + [B]­[Y]). Building on this classification, the subset of IL–IL pairs that are completely miscible and immiscible under experimental conditions and their corresponding COSMO-RS activity coefficients for the equimolar IL–IL mixture at 298.15 K are summarized in Table S3 of the Supporting Information. The experimental data show that IL–IL mixtures that have the same cation or the same anion usually have activity coefficients at equimolar compositions very close to one. This means that those mixtures behave almost ideally and are completely miscible. On the other hand, mixtures that contain different cations and different anions show a much wider range of activity coefficients. Some of these mixtures are miscible, while others are immiscible depending on the type of ions involved. Among the distinct cation–anion systems, mixtures that include imidazolium-based cations are mostly miscible, which suggests that this family of cations provides favorable interactions and good compatibility with a variety of anions. The available experimental data are limited, so the broader trends in cation- and anion-dependent miscibility are analyzed using a COSMO-RS-based large computational data set.

#### 3.1.2 Molecular Descriptor Generation

In COSMO-RS, each molecule is placed in an ideal conductor and its van der Waals surface is discretized into segments characterized by area Δ*A* and screening charge density σ.^18,20^ The distribution of surface area over σ defines the molecular σ-profile *p*_*i*_(σ) (reported here as a 50-bin histogram normalized to the unit area). The σ-profiles for cations and anions are generated individually with COSMO-RS 2023 using BP_TZVPD_FINE_23 parametrization, which improves the treatment of dispersion and hydrogen bonding relative to the original BP/TZVP set. Conformers are taken from the preoptimized COSMObase library; no additional geometry optimization is performed. Ion-pair descriptors are formed by point-wise summation of the cation and anion profiles to obtain a 50-bin representation of each pair. The summation of the cation and anion σ-profiles is considered as the σ-profiles of the IL.

### 3.2 ANN Model Development

To compute the activity coefficient of an IL–IL mixture, we trained an ANN model. Various computational models—including support vector machines (SVM), gradient-boosted and random forest, physics-informed neural networks, and other ML frameworks—have demonstrated strong performance in related contexts.^10,42–44^ In this work, we prefer ANNs because they provide the flexibility needed to capture the highly nonlinear relationships governing activity coefficients while producing continuous predictions that are directly reusable for downstream thermodynamic analysis and process-level applications beyond binary classification. The advantages of ANNs are particularly evident when a large experimental data set with a wide range of variables is available.^10,45^ In this work, an ANN architecture consisting of four layers (input layer, two hidden layers, and output layer) of a feedforward network is employed for building the model. The input layer consists of fundamental structural fragments such as sigma profiles of the ILs, along with the mole fraction of the binary mixture of ILs.

Let *j* ∈ {1,2} represent the two ILs (IL1, IL2), *i* ∈ {1,...,50} represent the σ-bins at {σ_*i*_}, *k* ∈ {1,...,*n*} represent the mixture IL–IL samples, and $x_{A}^{\left(k\right)}\in \left[0,1\right]$ be the mole fraction of IL_1_ for sample *k*, and

$$x^{\left(k\right)}=\left[p_{1}^{\left(k\right)}\left(\sigma_{1}\right),...,p_{1}^{\left(k\right)}\left(\sigma_{50}\right),p_{2}^{\left(k\right)}\left(\sigma_{1}\right),...,p_{2}^{\left(k\right)}\left(\sigma_{50}\right),x_{A}^{\left(k\right)}\right]\in R^{1\times 101}$$

$$p_{j}^{\left(k\right)}\left(\sigma_{i}\right)\Rightarrow P_{j}\in R^{n\times 50},,x_{A}^{\left(k\right)}\Rightarrow x_{A}\in R^{n\times 1}$$

9 $$X=\left[\begin{array}{c} x^{\left(1\right)}\\ x^{\left(2\right)}\\ \vdots \\ x^{\left(n\right)} \end{array}\right]\in R^{n\times 101}$$

We train a fully connected ANN implemented in PyTorch with two hidden layers of width *h* = 128 and ReLU activation, followed by a linear two-unit output layer that jointly predicts the targets [ln*γ̂*_mix_ *Ĥ* ^*E*^]. The ANN model takes $\left(X\in R^{n\times 101}\right)$ as input, calculates *Z*_1_ and *Z*_2_ in the hidden layer, and predicts the output set $\left(Ŷ\in R^{n\times 2}\right)$ as follows:

$$\begin{array}{llll} Z_{1} & =\;ReLU\left(XW_{1}^{T}+b_{1}^{T}\right), & W_{1}\in R^{128\times 101}, & b_{1}\in R^{128}\\ Z_{2} & =\;ReLU\left(Z_{1}W_{2}^{T}+b_{2}^{T}\right), & W_{2}\in R^{128\times 128}, & b_{2}\in R^{128}\\ Y & =\;Z_{2}W_{3}^{T}+b_{3}^{T}, & W_{3}\in R^{2\times 128}, & b_{3}\in R^{2} \end{array}$$ 10

The ANN model is trained using the Adam optimizer^46^ (learning rate: 10^–3^) while minimizing the mean squared error (MSE) over both output targets. Training proceeds for 40 epochs with mini-batches drawn from train_loader. Each iteration performs zeroing the gradients, performing a forward pass to obtain predictions, computing the MSE, back-propagating, and updating the parameters. At the end of each epoch, the network is switched to evaluation mode (model.eval­()). The training loss and validation loss are accumulated analogously to monitor the overfitting of the model. The output layer is consistent with the MSE objective for the continuous targets ln *γ̂*_mix_ and *Ĥ* ^*E*^. The predicted outputs of the model are interpreted as

11 $$Ŷ=\left[\ln{\mathrm{\gamma ̂}}_{\mathrm{mix}}{Ĥ}^{E}\right]$$

The labeled output $Y=\left[\ln\gamma_{\mathrm{mix}}H^{E}\right]\in R^{n\times 2}$ is computed using COSMO-RS. The ANN model is trained to minimize the mean squared error (MSE) between the predicted and reference values, which is given by

12 $$L_{\mathrm{MSE}}=\frac{1}{n}\overset{n}{\underset{k=1}{\sum }}∥{Ŷ}^{\left(k\right)}-Y^{\left(k\right)}{∥}_{2}^{2}$$

Before training, both the input features and the target data are normalized using the statistics of the training set. Each target is standardized separately by subtracting its mean and dividing it by its standard deviation. After the model generated predictions, the data are inverse-transformed to bring them back to the original physical scale so that they can be directly compared with the COSMO-RS reference values. Since the two outputs differ in scale, a weighted form of the MSE is tested to balance their influence during training:

$$L_{w}=\frac{1}{n}\overset{n}{\underset{k=1}{\sum }}\left(w_{1}{({\ln{\mathrm{\gamma ̂}}_{\mathrm{mix}}}^{\left(k\right)}-\ln\gamma_{\mathrm{mix}}^{\left(k\right)})}^{2}+w_{2}{({Ĥ}^{E,(k)}-H^{E,(k)})}^{2}\right)$$ 13

where *w*_1_ and *w*_2_ are positive constants that control the relative importance of the two terms. The model is optimized using mini-batches. Early stopping on a validation split is applied to prevent overfitting. Hidden width *h* and training hyperparameters are selected by a grid search based on validation performance.

### 3.3 Parity Plot Validation

The performance of the models is quantitatively evaluated using the coefficient of determination (*R* ^2^) and the root-mean-square error (RMSE):

$$\begin{array}{ll} \varepsilon_{T,k} & =\ln\gamma_{\mathrm{mix}}^{\mathrm{calc}}\left(x_{k},T\right)-\ln\gamma_{\mathrm{mix}}^{\mathrm{COSMO}}\left(x_{k},T\right),,S_{T}=\overset{N}{\underset{k=1}{\sum }}{[\ln{\;\gamma }_{\mathrm{mix}}^{\mathrm{COSMO}}(x_{k},T)-\overset{¯}{\ln{\;\gamma }_{\mathrm{mix}}^{\mathrm{COSMO}}}]}^{2}\\ R_{T}^{2} & =1-\frac{\overset{N}{\underset{k=1}{\sum }}\varepsilon_{T,k}^{2}}{S_{T}},,{\mathrm{RMSE}}_{T}=\sqrt{\frac{1}{N}\overset{N}{\underset{k=1}{\sum }}\varepsilon_{T,k}^{2}} \end{array}$$ 14

The outputs of the ML model (*γ̂* and *Ĥ* ^*E*^) and the values obtained from the temperature–correlation are plotted against COSMO-RS reference data. Points that fall near the identity line (*y* = *x*) indicate strong agreement between prediction and reference, demonstrating the high accuracy of both the ML model and the derived temperature-dependent correlation. The quantitative evaluation of model performance is based on the principle that a higher *R* ^2^ value and a lower RMSE indicate better predictive accuracy.

### 3.4 Binary Miscibility Classifier

A binary classification model is developed to predict the miscibility of IL–IL mixtures. The input features and ANN architecture of the binary classification model remain identical to those of the regression model (ReLU-ANN). However, the output activation function is replaced with a sigmoid function to enable binary classification. The model predicts a sigmoid output *ỹ* ∈ {0,1} from the previous input feature $X\in R^{n\times 101}$ , where [0] indicates an immiscible mixture and [1] indicates a miscible mixture. The binary classification ([0] for immiscible and [1] for miscible) simplifies the inherently complex phase behavior of IL–IL mixtures; however, it may not explicitly account for partial miscibility. At present, it remains unclear under what conditions IL–IL systems exhibit partial miscibility and whether such behavior constitutes a distinct thermodynamically stable intermediate state. A system may undergo a transition gradually between miscible and immiscible regimes. In this work, this transition is assumed to be effectively represented by a threshold composition. Consequently, systems exhibiting partial miscibility are conservatively classified as being immiscible.

The model is trained by minimizing the binary cross-entropy (BCE) loss function:

15 $$L_{\mathrm{BCE}}=-\frac{1}{N}\overset{N}{\underset{i=1}{\sum }}\left[y_{i}\;\ln\left({ŷ}_{i}\right)+\left(1-y_{i}\right)\;\ln\left(1-{ŷ}_{i}\right)\right]$$

where *y*_*i*_ and *ŷ*_*i*_ denote the true and predicted labels for sample *i*, respectively.

During training, the model parameters θ = {W, b} are iteratively updated in the direction of the negative gradient of the loss function:

16 $$\theta^{\left(t+1\right)}=\theta^{\left(t\right)}-\eta \nabla_{\theta }L_{\mathrm{BCE}}$$

where η is the learning rate and $\nabla_{\theta }L_{\mathrm{BCE}}$ denotes the gradient of the loss with respect to all trainable parameters.

The model generates probabilities between 0 and 1 for each class. At inference, the predicted probability *ŷ* is converted to a binary label *ỹ*. A mixture is classified as miscible [1] when the predicted probability exceeds τ = 0.5 and as immiscible [0] otherwise:

17 $$ỹ=\left\{ \begin{array}{ll} 1, & \mathrm{if}\;ŷ\geq \tau \\ 0, & \mathrm{if}\;ŷ<\tau \end{array}\right.$$

Confusion matrices are generated for both the experimentally validated IL–IL mixtures and the larger synthetic set of binary combinations. The classification criteria for the mixtures, which are not experimentally validated, are based on the activity coefficient of the mixture that is computed from the regression model (ReLU-ANN): When γ_mix_ ≤ 1.20, the system is considered miscible; otherwise, it is immiscible.

The performance of the classifier was evaluated in terms of precision (*P*), recall (*R*), F1-score (*F*_1_), and support. True positives, false positives, and false negatives are denoted as TP, FP, and FN, respectively. The corresponding expressions are

18 $$P=\frac{\mathrm{TP}}{\mathrm{TP}+\mathrm{FP}},,R=\frac{\mathrm{TP}}{\mathrm{TP}+\mathrm{FN}},,F_{1}=\frac{2PR}{P+R}$$

Precision represents the ratio of true positives to the total predicted positives and indicates the accuracy of the positive predictions. Recall measures the model’s ability to identify all positive instances, while the F1-score is the harmonic mean of precision and recall, balancing both metrics. Support corresponds to the number of actual occurrences of each class in the data set.

### 3.5 Temperature Correction

As the miscibility depends on the temperature, we estimate the activity coefficients beyond the reference temperature. We use eq 8 to calculate the activity coefficients at different temperatures, where the excess enthalpy is assumed to be constant for small temperature changes (Δ*T* ≈ ±30 K). We estimate activity coefficients at temperatures *T* ≠ *T*_0_ from reference data at *T*_0_ = 298.15 K and fixed composition *x* via a van’t Hoff-type relation.

### 3.6 Dimensionality Reduction

To explore qualitative patterns in IL combinations, t-distributed Stochastic Neighbor Embedding (t-SNE)^47^ is employed as a dimensionality-reduction and visualization tool. t-SNE operates on identity-based features constructed from the IL names rather than on molecular descriptors. Each binary pair is represented by its component identifiers (Cation_1_–Anion_1_) and (Cation_2_–Anion_2_), which are encoded as one-hot categorical vectors. This representation captures the co-occurrence of specific ions and highlights how mixtures that share a common cation or anion tend to cluster together in the reduced space. In this work, t-SNE is implemented using the scikit-learn^48^ Python library with default settings. This dimensionality-reduction technique projects the IL combinations into a 2D space where mixtures sharing a common cation or a common anion form distinct clusters, while systems with both different ions appear more widely dispersed. This visualization helps interpret the compositional similarity landscape and provides an intuitive view of how ionic identity influences miscibility trends.

## 4 Results and Discussion

### 4.1 Miscibility Predictions of IL–IL Mixtures

As discussed in the earlier section, the prediction of IL–IL miscibility is performed in two stages. First, given the σ-profiles of two ILs (combined 50-bin descriptors) and their IL–IL mixture mole fraction, the ANN model predicts the activity coefficient, ln *γ̂*_mix_, and the excess molar enthalpy, *Ĥ* ^*E*^. Next, the binary classifier classifies the given IL–IL mixture with a specific composition as miscible or immiscible. When training the ANN model, to ensure a fair evaluation, all experimentally reported IL–IL mixtures are first excluded from the data set. The remaining data set is divided into 64% for training, 16% for validation, and 20% for testing, and the excluded experimental data are then appended to the test set as an external benchmark. To mitigate data leakage, the data set was split at the system level, ensuring that identical IL–IL combinations were not shared between the training and test sets. While this approach supports robust model evaluation, the predictive accuracy may be reduced for IL chemistries or subclasses that are sparsely represented or absent from the training data.

For the first part, the parity plots in Figure 3A–F show that predictions for both ln *γ̂*_mix_ and *Ĥ* ^*E*^ follow the diagonal *y* = *x* line closely across the training, validation, and testing subsets, with *R* ^2^ > 0.90 and low errors (RMSE ≈ 0.016–0.017 for ln *γ̂*_mix_ and ≈0.0145–0.051 for *Ĥ* ^*E*^). The slopes near unity and the intercepts close to zero for all plots confirm that ReLU-ANN predicts the target properties well. The red dots represent the experimental mixtures excluded from training, which also fall near the diagonal, confirming that the model generalizes well to the unseen experimental data. This demonstrates that the ANN model captures the underlying thermodynamic relationships governing the IL–IL interactions. The evolution of the loss function (Figure S2 of the Supporting Information) also demonstrates that the model performs consistently on both the training and validation data sets, indicating good convergence without signs of overfitting. Since activity coefficients indicate miscibility behavior, the strong predictive performance makes this model a useful tool for rapid screening of IL–IL mixtures. Accordingly, the model is intended as a high-level screening tool, and caution should be exercised when extrapolating to chemically distinct IL systems.

**Figure 3:**
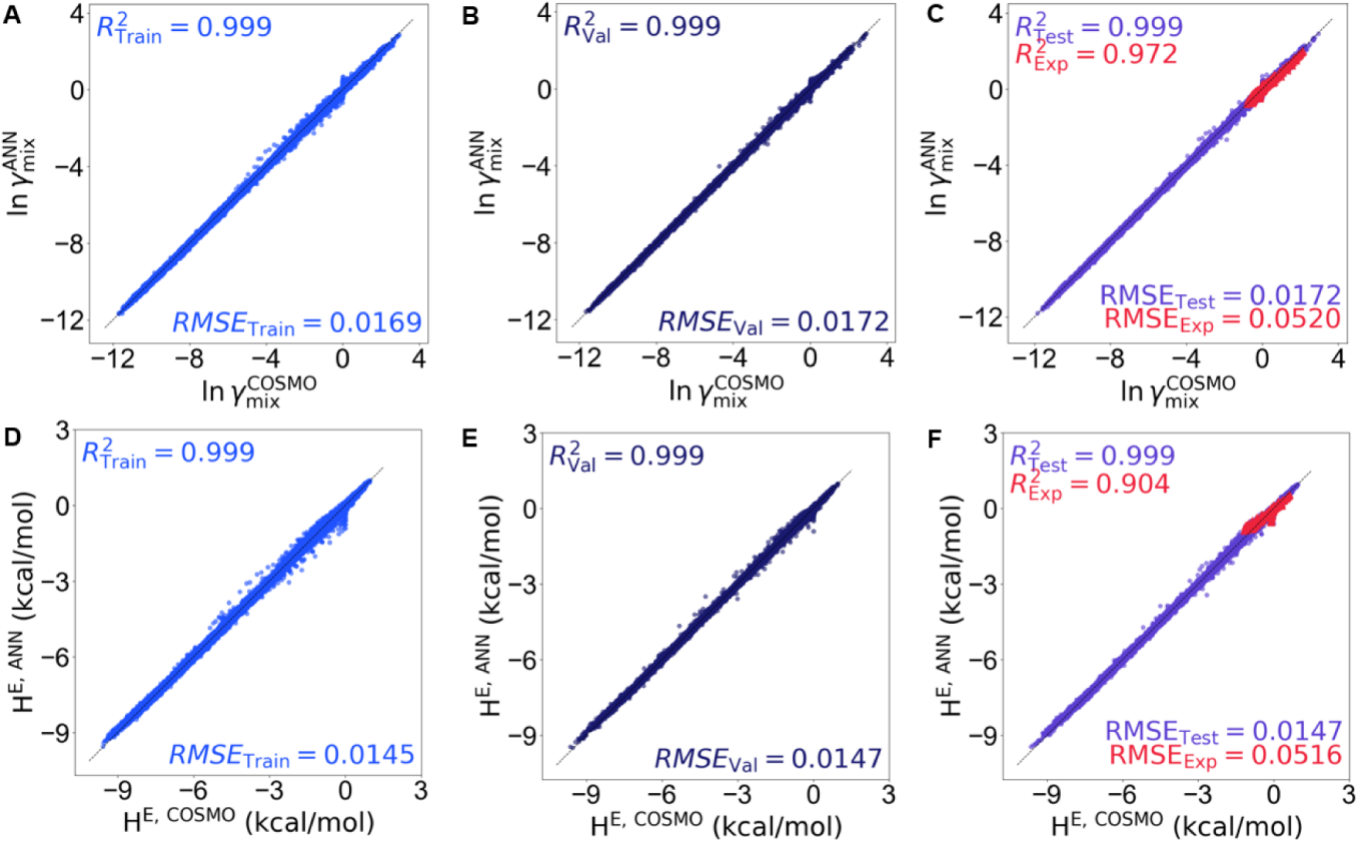
ANN performance for (A–C) ln γ_mix_ and (D–F) *H^E^* during training, validation, and testing, respectively. Red dots in the testing panels indicate experimental binary mixtures that are excluded from training and used for validation. All show *R* ^2^ > 0.90 and low RMSE values (≈0.016–0.017 for ln *γ̂*_mix_; ≈0.014–0.051 for *Ĥ^E^*), confirming excellent predictive accuracy and generalization of the model.

To predict beyond the reference temperature, the activity coefficients of the IL–IL mixture are estimated using the derived van’t Hoff-type temperature correlation. The reliability of this temperature-correction approach is examined by comparing the calculated activity coefficients with direct COSMO-RS predictions at 290.15, 305.15, and 310.15 K. The corresponding parity plots (Figure 4) show the correlation between $\gamma_{\mathrm{mix}}^{\mathrm{calc}}\left(x,T\right)$ obtained from the van’t Hoff relation and $\gamma_{\mathrm{mix}}^{\mathrm{COSMO}}\left(x,T\right)$ from independent COSMO-RS calculations. The data points cluster tightly around the diagonal line for all three temperatures, demonstrating excellent quantitative agreement and confirming the correlation accuracy. The very high *R* ^2^ and low RMSE across the examined temperature range further demonstrate that a constant excess enthalpy is a reasonable approximation for small temperature deviations (Δ*T*).

**Figure 4:**
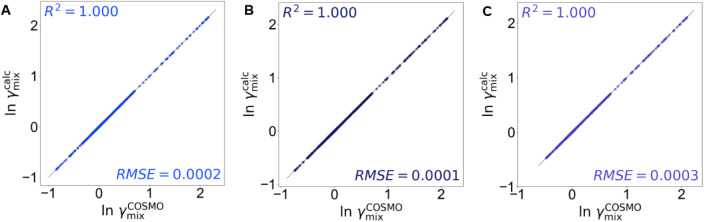
Activity coefficients obtained from the van’t Hoff-type temperature correlation, $\gamma_{\mathrm{mix}}^{\mathrm{calc}}\left(x,T\right)$ , and those predicted directly by COSMO-RS, $\gamma_{\mathrm{mix}}^{\mathrm{COSMO}}\left(x,T\right)$ , at (A) 290.15, (B) 305.15, and (C) 310.15 K. The excellent correlation across all temperatures demonstrates that the constant-excess enthalpy assumption provides a reliable approximation for small temperature deviations.

By coupling this correction with the ANN regression model, γ_mix_ and *H* ^*E*^ values can be efficiently updated across realistic operating temperatures without rerunning high-cost COSMO-RS simulations. This integration allows temperature effects to be accounted for during miscibility classification and extends the framework’s predictive scope from a single reference state to a continuous temperature domain relevant for practical solvent and separation process design.

After the fast prediction of the activity coefficients, a separate ANN is trained using the same input features and model architecture but with a sigmoid activation function in the output layer to classify each IL–IL mixture as miscible or immiscible. For the computationally generated data set, the classification labels are defined using the activity coefficient γ_mix_ threshold. The mixtures with γ_mix_ ≤ 1.20 are labeled as miscible, while those with higher values are labeled as immiscible. These labels are then used to train the binary classifier.

The performance of the model is evaluated on two independent data sets. First, the experimentally known miscibility data containing 77 IL–IL mixtures at various compositions (1,662 miscible and 209 immiscible cases) is used to assess the model’s predictive reliability. As shown in Figure 5A, the confusion matrix indicates excellent agreement between the ANN predictions and experimental observations with only a few minor deviations. The corresponding metrics in Table 1A show precision, recall, and F_1_-scores close to one for both classes, confirming that the network accurately distinguishes between miscible and immiscible systems.

**Table 1:** Performance Metrics of the Binary Classification Model Evaluated on (A) the Experimentally Validated IL–IL Mixtures and (B) the Computationally Generated Data set Labeled Using the *γ*_mix_ = 1.20 Criterion

Data set	Class	Precision	Recall	F_1_-score	Support
(A) Experimental	Immiscible	1.00	0.99	0.99	209
	Miscible	1.00	1.00	1.00	1,682
(B) Computational	Immiscible	0.90	0.99	0.95	64,536
	Miscible	1.00	0.99	1.00	916,766

**Figure 5:**
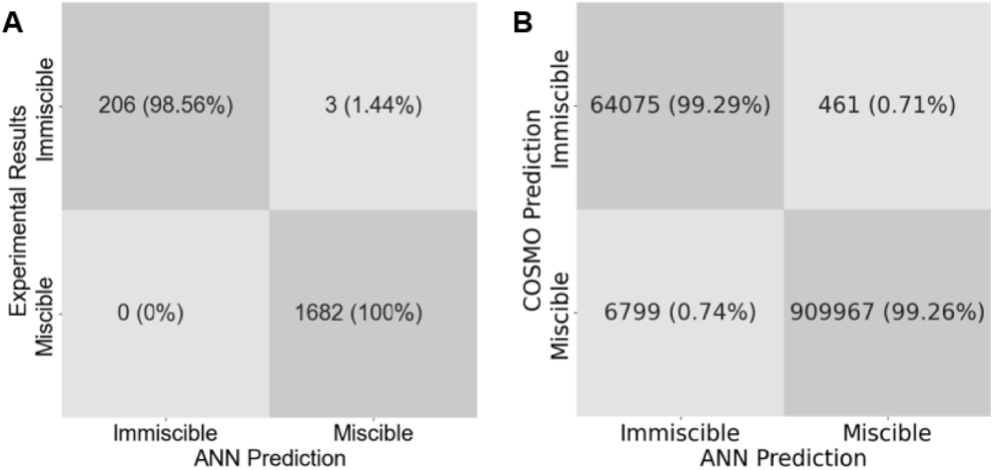
Confusion matrices for the binary classification model predicting miscible and immiscible IL–IL mixtures. Results shown are for experimentally validated IL–IL mixtures (A) and for hypothetical IL–IL mixtures (B) at different compositions.

The model is then applied to the test set composed of 20% of the large computational data set, which includes all possible combinations of common cations and anions. As shown in Figure 5B, the confusion matrix demonstrates that most systems are correctly classified, relative to their COSMO-RS-based labels. The performance metrics summarized in Table 1B show a slightly lower precision for the immiscible class, which can be attributed to the smaller number of immiscible samples and the presence of borderline cases near the γ_mix_ = 1.20 cutoff. Such cases are expected since miscibility transitions are continuous rather than sharply defined.

Overall, the binary classification model shows strong consistency with both the experimental and computational results. The loss function curve in Figure S3 (Supporting Information) shows that the training and validation losses track closely with no sign of divergence, demonstrating no overfitting. Therefore, we have a reliable and efficient approach for predicting miscibility directly from molecular identity features, eliminating the need for separate regression steps or additional COSMO-RS calculations.

### 4.2 Miscibility and Molecular Structural Similarity

A σ-profile represents the probability distribution of molecular surface charge density (σ) obtained from quantum chemical COSMO calculations.^18^ The degree of overlap between the σ-profiles of the two ionic liquids provides a qualitative measure of their mutual compatibility. When two ILs exhibit highly similar charge-distribution patterns, they tend to form stable, miscible mixtures, consistent with the classical *like dissolves like* principle.^21^ In contrast, pronounced differences between their σ-profiles reflect weaker electrostatic and hydrogen-bonding interactions, which often lead to immiscibility.^20^

Figure 6 shows representative examples of σ-profiles for (A) IL mixtures sharing a similar cation, (B) IL mixtures sharing a similar anion, (C) IL mixtures with both different cations and anions, and (D) an immiscible IL pair. The two vertical dashed lines denote three characteristic surface regions: the left region corresponds to hydrogen-bond acceptor sites; the central region corresponds to nonpolar interactions; and the right region corresponds to hydrogen-bond donor sites. Miscible ILs generally display comparable charge-density distributions across these regions, whereas immiscible IL mixtures appear to show clear mismatches, particularly in the hydrogen-bonding regions.

**Figure 6:**
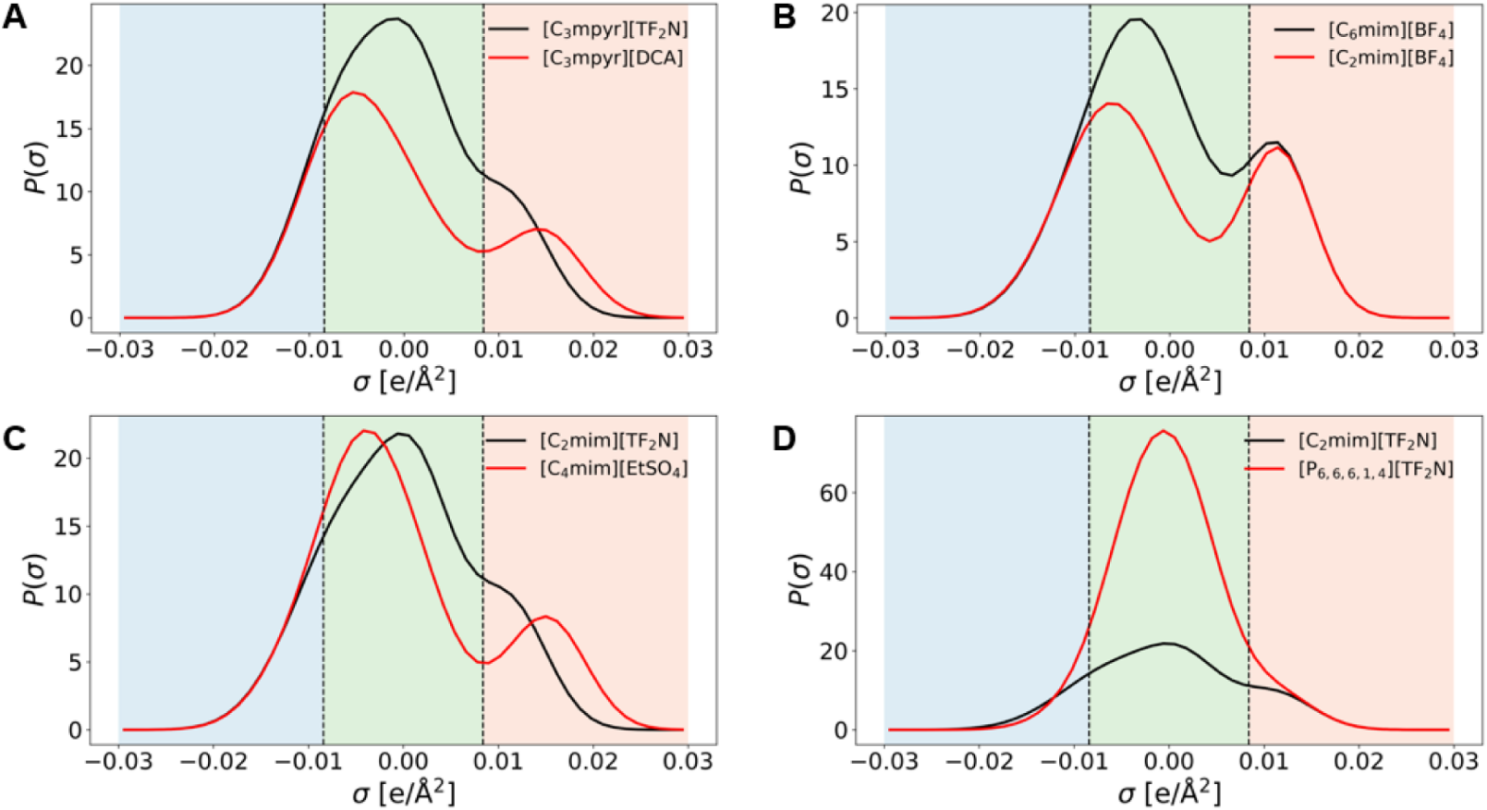
Representative σ-profiles illustrating different types of IL–IL mixtures. (A) Mixtures with a similar cation, (B) mixtures with a similar anion, (C) mixtures containing both different cations and anions, and (D) an immiscible combination. The vertical dashed lines divide the surface charge density into hydrogen-bond acceptor (left), nonpolar (center), and hydrogen-bond donor (right) regions. Greater overlap of the σ-profiles corresponds to stronger molecular compatibility and higher miscibility, whereas larger differences indicate a reduced intermolecular affinity and potential immiscibility.

Although a visual comparison of σ-profiles offers a straightforward qualitative guide, it cannot alone provide quantitative miscibility predictions. Accurate assessment also requires consideration of molecular interactions, thermodynamic behavior, and composition effects. Nevertheless, the σ-profile analysis remains a useful first step for screening ionic liquid combinations and guiding the rational design of working fluids for energy and separation processes.

To visualize the distribution of ionic liquid mixtures by their molecular identities, a name-based t-SNE embedding is generated for the large computational data set. Each mixture is represented by its component identifiers (Cation_1_–Anion_1_) and (Cation_2_–Anion_2_), which are one-hot-encoded and projected into two dimensions for visualization purposes. The color scale corresponds to the COSMO-RS predicted activity coefficient γ_mix_ between 0 to 2, where mixtures with γ_mix_ ≤ 1.20 are considered miscible.

Figure 7 shows the embeddings for three subsets: (A) mixtures with the same cation, (B) mixtures with the same anion, and (C) mixtures with both different cations and anions. All three embeddings are generated by using identical t-SNE parameters and visualization settings, and differences in appearance arise primarily from the chemical diversity of each subset. For systems sharing a common cation, the points form distinct compact clusters with mostly low γ_mix_ values (blue–green region), indicating that these mixtures are generally miscible. When the anion is fixed and the cation varies, the color distribution becomes broader, reflecting that miscibility depends more strongly on the cation structure. For mixtures where both ions differ, the data occupy a more continuous and scattered space with a wide range of γ_mix_ values, from fully miscible to clearly immiscible. This trend suggests that molecular identity, particularly cation–anion pairing, plays a central role in determining miscibility behavior across the IL–IL mixture space.

**Figure 7:**
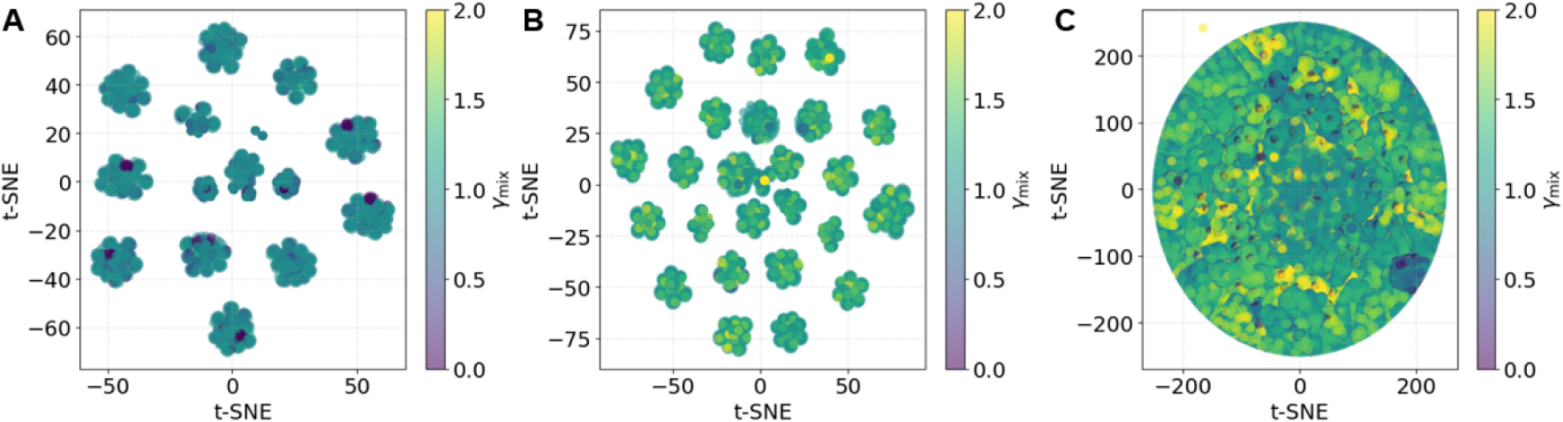
Two-dimensional t-SNE embeddings of ionic liquid mixtures colored by the COSMO-RS predicted activity coefficient, γ_mix_ (color scale 0–2; mixtures with γ_mix_ ≤ 1.20 are considered miscible). (A) Mixtures sharing a common cation form compact clusters with mostly low γ_mix_ values, indicating strong miscibility. (B) Mixtures sharing a common anion display a broader spread in color, showing greater variation in miscibility as different cations are introduced. (C) Mixtures with both distinct cations and anions exhibit a wide range of γ_mix_ values, revealing a complex balance of interactions and a higher likelihood of immiscibility.

### 4.3 Experimental Confirmation of Miscibility Predictions

To test and confirm the predictive capability of our framework, we also experimentally measured the miscibility of several binary IL–IL mixtures. Specifically, we tested six binary mixtures by considering species from a pool of four ILs: [C_2_mim]­[Tf_2_N], [C_2_mim]­[SCN], [C_4_mim]­[Tf_2_N], and [C_4_mim]­[SCN]. These generate six unique binary combinations, which are prepared at a 50/50 wt % composition and tested experimentally.

The three ILs [C_2_mim]­[Tf_2_N] (99.5 wt % purity), [C_4_mim]­[Tf_2_N] (99.5 wt % purity), and [C_2_mim]­[SCN] (98 wt % purity) are purchased from IoLiTec GmbH, while [C_4_mim]­[SCN] (95 wt % purity) is purchased from Fluka. To minimize the presence of water and volatile components, a careful drying and mixing protocol is followed based on the procedure described by Tomé et al.^49^ The pure IL samples are first dried at 40 °C under vacuum (≈10^–3^ kPa) for 4 days prior to mixing. After drying, the sample vials are immediately sealed and transferred into a nitrogen-purged glovebox to avoid moisture uptake during sample preparation. Each IL mixture is then weighed to obtain a 50/50 wt % composition and mixed on a stir plate for 30 min. After mixing, the vials are returned to the vacuum oven at 40 °C under vacuum (≈10^–3^ kPa) for an additional 2 days. Figure 8A shows a schematic of the overall procedure.

**Figure 8:**
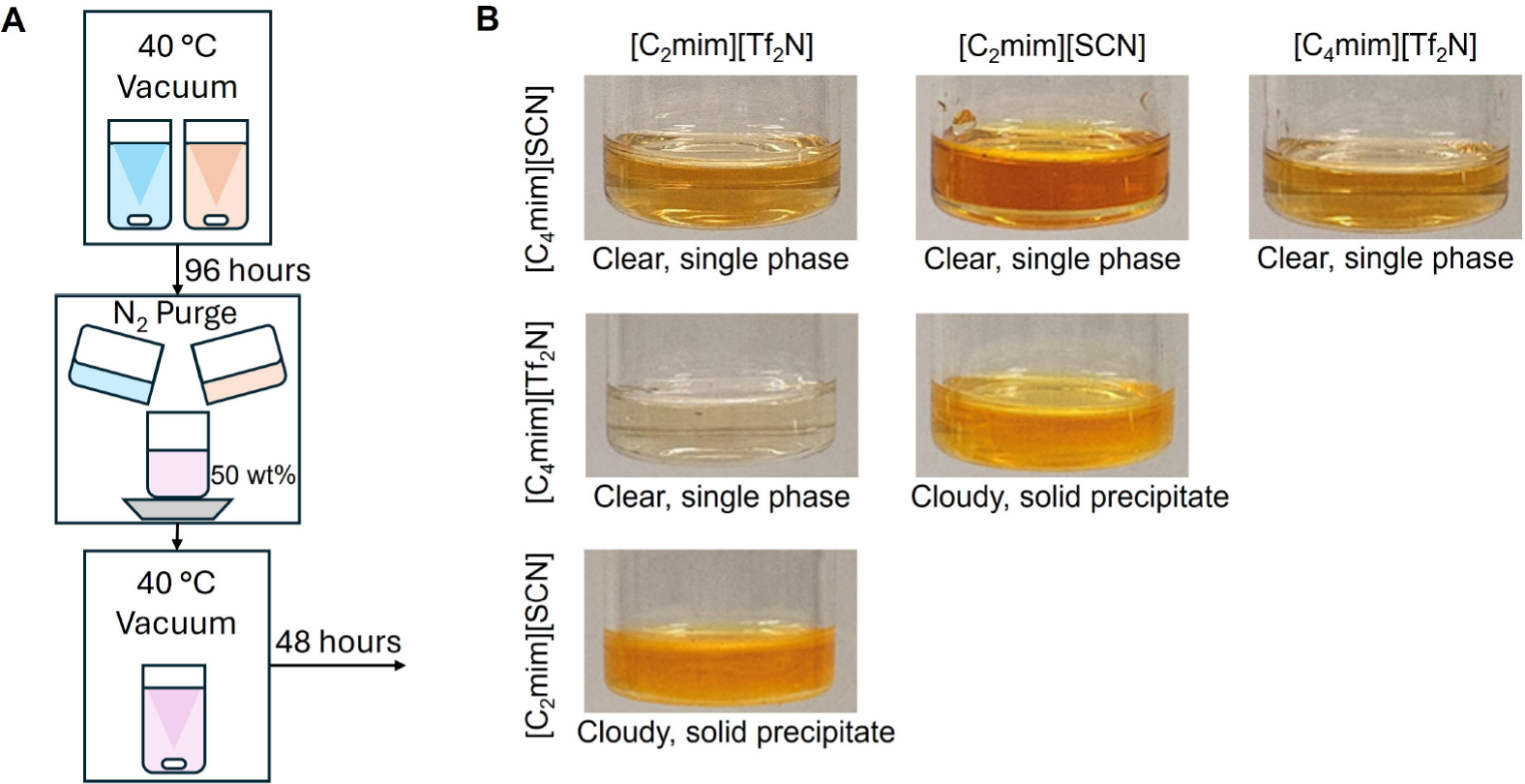
Sample preparation method (A) and photographs of the prepared IL–IL mixtures (B) used for experimental miscibility validation. [C_2_mim]­[Tf_2_N] + [C_2_mim]­[SCN] and [C_4_mim]­[Tf_2_N] + [C_2_mim]­[SCN] mixtures exhibit cloudiness and the formation of a solid precipitate that settles to the bottom of the container. All other mixtures produced a single clear phase.

The computational framework predicts that all six mixtures should be fully miscible at this composition. Immediately after mixing, all of the IL–IL mixtures appear as clear single-phase liquids. This confirms that our computational framework is able to make the correct predictions.

The mixtures are also monitored over time to confirm phase stability. Interestingly, after approximately 24 h, two out of the six mixtures ([C_2_mim]­[Tf_2_N] + [C_2_mim]­[SCN] and [C_4_mim]­[Tf_2_N] + [C_2_mim]­[SCN]) become slightly cloudy. After 48 h, visible solid precipitates are observed settling at the bottom of the sample vials for these two systems. The remaining mixtures show no signs of secondary phase formation during the observation period and remain visually clear. Figure 8B shows the experimental samples of the six IL–IL mixtures.

It is important to note that IL samples are rarely perfectly pure liquids. The [C_2_mim]­[SCN] sample used in these experiments has a reported purity of 98 wt %, meaning that the remaining 2% may contain residual water, trace impurities, or unreacted synthesis precursors. These trace components may react with the second ionic liquid in the mixture (in this case, [C_2_mim]­[Tf_2_N]), leading to the formation of solid products. One possible mechanism is an ion-exchange reaction between an ionic liquid and residual precursors present in the sample. The computational model developed in this work does not account for impurities, precursors, or moisture content. Therefore, even if a binary IL pair is predicted to be miscible for the pure components, precipitates may still form experimentally depending on the chemical history and purity of the samples.

A previous experimental study of the [C_2_mim]­[Tf_2_N] + [C_2_mim]­[SCN] mixture reported by Tomé et al.^49^ did not observe precipitation despite using a similar experimental procedure. This can be attributed to the [C_2_mim]­[SCN] IL being obtained from a different supplier with a reported purity of 95 wt %. Differences in synthesis routes or precursor residues between suppliers may influence the likelihood of secondary reactions and solid formation in these systems.

Overall, the experimental observations are largely consistent with the miscibility trends predicted by the computational model. The miscibility results are summarized in Table 2. Most of the tested IL pairs remain fully miscible under the experimental conditions, while the two systems showing precipitation are likely influenced by impurities or residual precursors rather than the true immiscibility of the pure ILs.

**Table 2:** Experimental Miscibility of the Selected IL–IL Mixtures^a^

IL Name	[C_2_mim][Tf_2_N]	[C_2_mim][SCN]	[C_4_mim][Tf_2_N]
[C_4_mim][SCN]	Miscible	Miscible	Miscible
[C_4_mim][Tf_2_N]	Miscible	Solid precipitate	
[C_2_mim][SCN]	Solid precipitate		

a The computational framework predicts that all are miscible.

## 5 Conclusions

We put forward an integrated framework that combines hybrid quantum chemical and thermodynamic calculations in the COSMO-RS with data-driven machine learning to predict the miscibility of binary mixtures of ionic liquids. Mixtures with components having similar trends in surface charge densities, which can be captured in σ-profiles, generally have activity coefficients near unity and remain fully miscible, whereas larger deviations in surface charge density correspond to immiscibility. Our results indicate that a ReLU-based ANN model is sufficient to predict both ln γ_mix_ and *H* ^*E*^ with high accuracy when compared with the COSMO-RS predicted values. A binary classification model based on these predicted thermodynamic variables can successfully distinguish miscible from immiscible mixtures. A van’t Hoff-type temperature correlation extends the predictions beyond the reference temperature of 298.15 K, accurately predicting activity coefficients as corroborated by COSMO-RS results. Together, they form a comprehensive workflow that links molecular descriptors, thermodynamic quantities, and macroscopic phase behavior to predict the possibility of a binary mixed solvent based on different ionic liquids. To further assess the reliability of the model predictions, experimental miscibility tests were performed for selected IL–IL mixtures. The experimental observations are largely consistent with the predicted miscibility trends, with most tested mixtures remaining fully miscible under the experimental conditions. In a few cases, solid precipitation was observed after an extended time, which is likely influenced by impurities or residual precursors in the ionic liquid samples rather than the intrinsic immiscibility of the pure components. These experimental results provide additional support for the predictive capability of the proposed framework and highlight the importance of sample purity in practical miscibility studies. The framework enables rapid, data-driven screening of ionic liquid mixtures, reducing reliance on costly experiments and high-fidelity simulations. Beyond its practical use for solvent selection, the results also help clarify how cation–anion identity and charge-distribution similarity govern miscibility trends.

## 6 Data and Software Availability

Data sets, together with ML model parameters and complete source code, are publicly available at the following link: https://github.com/chenahid/Ionic-Liquid-Miscibility/releases/tag/Miscibility

## Supplementary Material



## Notes

Comprehensive lists of selected ions, experimentally reported IL–IL miscible and immiscible systems, activity coefficients at 298.15 K, ML model training details, and optimized process conditions for varying IL compositions are available in the Supporting Information (PDF)
