# Supplementary material for: Predicting Miscibility of Ionic Liquids in Binary Mixtures
Source: J Chem Inf Model. 2026 Apr 14;66(8):4427–38. doi: 10.1021/acs.jcim.6c00050 (PMC13126627; doi:10.1021/acs.jcim.6c00050)
Supplement: Supplementary file 1 [file ci6c00050_si_001.pdf]

Predicting Miscibility of Ionic Liquids in Binary Mixtures

Ahaduzzaman Nahid[1], Ashfaq Iftakher[1], Puneeth Ganesh Babu Agoram[1], Luke W. Wallisch[2], Abby N. Harders[2], Kalin R. Baca [2], and M. M. Faruque Hasan[1,3,*]

[1]Artie McFerrin Department of Chemical Engineering, Texas A&M University, College Station, TX 77843-3122, USA.

[2]Icorium Engineering Company, Lawrence, KS 66047, USA

[3]Texas A&M Energy Institute, Texas A&M University, College Station, TX, 77843, USA.

Contents

S1 Selected Ions for Binary Ionic Liquid Mixture Analysis **S3**

S2 Experimentally Miscible and Immiscible IL–IL Mixtures **S4**

S3 ML Models for Predicting IL–IL Miscibility **S7**

S4 Optimized Process Conditions Across IL–IL Compositions **S8**

List of Tables

S1 List of selected cations and anions for binary liquid mixture analysis. S3

S2 List of cations and anions with their abbreviations for experimentally miscible and immiscible IL-IL mixtures. S4

S3 Activity coefficients (γ_{mix}) for equimolar ionic liquid (IL) pairs at 298.15 K, grouped by miscible (Same Cation, Same Anion, Distinct Cation & Anion) and immiscible mixture. S5

S4 Optimized process variables, product purities, and total duty at different [C_2mim][TF_2N] compositions in [C_2mim][TF_2N]+[C_2mim][SCN] mixture. . S9

List of Figures

S1 Training and validation loss curves over 40 epochs for the regression model. S7
S2 Training and validation loss curves over 40 epochs for the binary classification model. S8

S1 Selected Ions for Binary Ionic Liquid Mixture Analysis

A total of 32 commonly used cations and 32 anions have been considered in this study to construct a comprehensive set of ionic liquids (ILs). By combining these cations and anions, a diverse binary IL–IL mixtures is generated. The complete list of cations and anions included in the screening is presented in Table S1.

Table S1: List of selected cations and anions for binary liquid mixture analysis.

SL	Cation Name	SL	Anion Name
1	1-methyl-1-propylpyrrolidinium	1	bis(trifluoromethanesulfonyl)imide
2	1,1-dipropyl-pyrrolidinium	2	tetrafluoroborate
3	1-butyl-1-methyl-pyrrolidinium	3	thiocyanate
4	1-hexyl-1-methyl-pyrrolidinium	4	ethylsulfate
5	1-ethyl-pyridinium	5	methanesulfonate
6	1-butyl-3-methyl-pyridinium	6	chloride
7	1-butyl-pyridinium	7	bromide
8	3-methyl-1-propylpyridinium	8	iodide
9	3-methyl-1-octyl-pyridinium	9	picrate
10	1-methyl-3-methyl-imidazolium	10	methylsulfate
11	1-ethyl-3-methyl-imidazolium	11	nitrate
12	1-propyl-3-methyl-imidazolium	12	acetate
13	1-butyl-3-methyl-imidazolium	13	dihydrogen-phosphate
14	1-pentyl-3-methyl-imidazolium	14	benzenesulfonate
15	1-hexyl-3-methyl-imidazolium	15	toluene-4-sulfonate
16	1-octyl-3-methyl-imidazolium	16	trifluoromethane-sulfonate
17	1-butyl-2,3-dimethyl-imidazolium	17	methanesulfonate
18	1-methyl-3-nonylimidazolium	18	dicyanamide
19	tetra-n-butylphosphonium	19	tricyanomethane
20	tetrapentylphosphonium	20	hexafluorophosphate
21	trihexyl-tetradecyl-phosphonium	21	dimethylphosphate
22	tetraphenyl-phosphonium	22	hydroxide
23	tetra-n-butylammonium	23	hydrogen sulfate
24	tetramethylammonium	24	tetrachloroborate
25	1-methyl-1-propylpiperidinium	25	sulfate
26	1-(3-methoxypropyl)-1-methylpyrrolidinium	26	tetrachloroferrate(iii)_hextuplet
27	di[n-di(2-methoxyethyl)]]-n-dimethyl-guanidinium	27	chloroform
28	1-(2-hydroxyethyl)-3-methylimidazolium	28	1,2,3-triazole
29	1-decyl-3-methyl-imidazolium	29	1,2,4-triazole
30	dimethylamine	30	formate
31	ethanolamine	31	tetrachlorogallate
32	methylamine	32	tetrazole

S2 Experimentally Miscible and Immiscible IL–IL Mixtures

A comprehensive set of experimentally reported miscible and immiscible IL–IL mixtures has been compiled based on an extensive review of peer-reviewed literature. The names of the ionic liquids, along with their corresponding abbreviations, are provided in Table S2.

For each IL–IL pair, the activity coefficients are computed across multiple compositions using the COSMO-RS quantum computing software. These computations allow the identification of miscibility threshold between ionic liquid pairs. The activity coefficients of the equimolar IL–IL mixtures, which serve as key indicators of miscibility in this work, are summarized in Table S3.

Table S2: List of cations and anions with their abbreviations for experimentally miscible and immiscible IL-IL mixtures.

SL	Cation Name	Cation Abbreviation	SL	Anion Name	Anion Abbreviation
1	1-methyl-3-methylimidazolium	[C_1mim]	1	bis((trifluoromethyl)sulfonyl)imide	[Tf_2N]
2	1-ethyl-3-methyl-imidazolium	[C_2mim]	2	tetrafluoroborate	[BF_4]
3	1-propyl-3-methyl-imidazolium	[C_3mim]	3	ethylsulfate	[$EtSO_4$]
4	1-butyl-3-methyl-imidazolium	[C_4mim]	4	thiocyanate	[SCN]
5	1-pentyl-3-methyl-imidazolium	[C_5mim]	5	dicyanamide	[DCA]
6	1-hexyl-3-methyl-imidazolium	[C_6mim]	6	bromide	[Br]
7	1-octyl-3-methyl-imidazolium	[C_8mim]	7	methylsulfate	[$MeSO_4$]
8	1-decyl-3-methyl-imidazolium	[C_{10}mim]	8	hexafluorophosphate	[PF_6]
9	1-butyl-2,3-dimethyl-imidazolium	[C_4C_1mim]	9	methanesulfonate	[$MeSO_3$]
10	1-(2-hydroxyethyl)-3-methylimidazolium	[HC_2mim]	10	tricyanomethane	[TCM]
11	1-methyl-1-propylpyrrolidinium	[C_3mpyr]	11	trifluoromethane-sulfonate	[$TfMeSO_3$]
12	1-butyl-1-methyl-pyrrolidinium	[C_4mpyr]	12	acetate	[Ac]
13	1-hexyl-1-methyl-pyrrolidinium	[C_6mpyr]	13	dimethylphosphate	[$DMePO_4$]
14	1-butyl-3-methylpyridinium	[B_3mpy]	14	chloride	[Cl]
15	3-methyl-1-octyl-pyridinium	[O_3mpy]	15	formate	[CHO_2]
16	1,1-dipropyl-pyrrolidinium	[Prmpyr]	16	nitrate	[NO_3]
17	1-ethyl-pyridinium	[C_2py]	17	hydrogen sulfate	[HSO_4]
18	1-butyl-pyridinium	[Bpy]	18	heptachlorodialuminate	[Al_2Cl_7]
19	1-ethyl-pyridinium	[Epy]	19	tetrachloroaluminate	[$AlCl_4$]
20	ethanolamine	[EthAM]	20	tetrachlorogallate	[$GaCl_4$]
21	methylamine	[MeAM]	21	1,2,3-triazole	[3-Triz]
22	dimethylamine	[DMeAM]	22	1,2,4-triazole	[4-Triz]
23	ethanolamine	[EthAM]	23	tetrazole	[tzole]
24	1-methyl-1-propylpiperidinium	[C_3mpip]	24	bis(pentafluoroethylsulfonyl)amide	[BETI]
25	trihexyl-tetradecyl-phosphonium	[$P_{6,6,6,1,4}$]			
26	tetra-n-butylphosphonium	[$P_{4,4,4,4}$]			
27	tetraphenyl-phosphonium	[PPh_4]			
28	tetramethylammonium	[NMe_4]			

Table S3: Activity coefficients (γ_{mix}) for equimolar ionic liquid (IL) pairs at 298.15 K, grouped by miscible (Same Cation, Same Anion, Distinct Cation & Anion) and immiscible mixture.

**Mixture Type**	**SL**	**IL1**		**IL2**		γ_{mix}	**Reference**
		**Cation1**	**Anion1**	**Cation2**	**Anion2**		
	1	[C$_3$mpyr]	[TF$_2$N]	[C$_3$mpyr]	[DCA]	0.92	1
	2	[B$_3$mpy]	[BF$_4$]	[B$_3$mpy]	[DCA]	1.01	2
	3	[C$_5$mim]	[TF$_2$N]	[C$_5$mim]	[Br]	0.73	3
	4	[C$_2$mim]	[TF$_2$N]	[C$_2$mim]	[EtSO$_4$]	0.90	4
	5	[C$_2$mim]	[BF$_4$]	[C$_2$mim]	[DCA]	1.02	5
	6	[C$_4$mim]	[BF$_4$]	[C$_4$mim]	[MeSO$_4$]	1.01	6
	7	[C$_4$mim]	[BF$_4$]	[C$_4$mim]	[PF$_6$]	0.96	6
	8	[C$_2$mim]	[EtSO$_4$]	[C$_2$mim]	[MeSO$_4$]	1.00	7
	9	[C$_2$mim]	[EtSO$_4$]	[C$_2$mim]	[MeSO$_3$]	0.96	7
	10	[Bpy]	[BF$_4$]	[Bpy]	[TF$_2$N]	1.00	8
	11	[C$_2$mim]	[SCN]	[C$_2$mim]	[DCA]	0.99	9
	12	[C$_4$mim]	[TF$_2$N]	[C$_4$mim]	[SCN]	0.97	10
	13	[C$_4$mim]	[TF$_2$N]	[C$_4$mim]	[DCA]	0.94	10
	14	[C$_4$mim]	[TF$_2$N]	[C$_4$mim]	[TCM]	0.96	10
**Same Cation ILs (Miscible)**	15	[C$_4$mim]	[TF$_2$N]	[C$_4$mim]	[BF$_4$]	1.00	10
	16	[C$_4$mim]	[TF$_2$N]	[C$_4$mim]	[PF$_6$]	1.07	10
	17	[C$_4$mim]	[TF$_2$N]	[C$_4$mim]	[TfMeSO$_3$]	0.98	10
	18	[C$_4$mim]	[TF$_2$N]	[C$_4$mim]	[Ac]	0.46	10
	19	[C$_4$mim]	[TF$_2$N]	[C$_4$mim]	[DMePO$_4$]	0.77	10
	20	[C$_4$mim]	[Cl]	[C$_4$mim]	[TF$_2$N]	0.62	11
	21	[C$_4$mim]	[PF$_6$]	[C$_4$mim]	[TfMeSO$_3$]	1.01	11
	22	[C$_4$mim]	[BF$_4$]	[C$_4$mim]	[Ac]	0.59	11
	23	[C$_6$mim]	[Cl]	[C$_6$mim]	[PF$_6$]	0.74	11
	24	[C$_8$mim]	[Cl]	[C$_8$mim]	[BF$_4$]	0.89	11
	25	[C$_2$mim]	[Cl]	[C$_2$mim]	[GaCl$_4$]	0.46	11
	26	[C$_4$mim]	[NO$_3$]	[C$_4$mim]	[MeSO$_3$]	0.99	12
	27	[C$_4$mim]	[MeSO$_3$]	[C$_4$mim]	[Cl]	0.94	12
	28	[C$_4$mim]	[NO$_3$]	[C$_4$mim]	[Cl]	0.94	12
	29	[C$_6$mim]	[Cl]	[C$_6$mim]	[BF$_4$]	0.87	13
	30	[P66614]	[3-Triz]	[P66614]	[TF$_2$N]	0.87	12
	31	[C$_3$mpyr]	[TF$_2$N]	[C$_4$mpyr]	[TF$_2$N]	1.00	1
	32	[B$_3$mpy]	[BF$_4$]	[O$_3$mpy]	[BF$_4$]	1.02	2
	33	[C$_6$mim]	[BF$_4$]	[C$_2$mim]	[BF$_4$]	1.05	6
	34	[C$_6$mim]	[BF$_4$]	[C$_4$mim]	[BF$_4$]	1.01	6
	35	[C$_4$mim]	[TF$_2$N]	[C$_4$C$_1$mim]	[TF$_2$N]	1.00	14
	36	[C$_4$mim]	[TF$_2$N]	[C$_3$mpip]	[TF$_2$N]	0.99	15
	37	[C$_3$mpyr]	[TF$_2$N]	[C$_6$mpyr]	[TF$_2$N]	1.01	16
	38	[C$_3$mpyr]	[TF$_2$N]	[C$_2$mim]	[TF$_2$N]	1.00	16
	39	[C$_3$mim]	[BF$_4$]	[C$_6$mim]	[BF$_4$]	1.03	11
	40	[C$_2$mim]	[BF$_4$]	[C$_3$mim]	[BF$_4$]	1.01	11

Same Anion ILs (Miscible)

Mixture Type	SL	IL1		IL2		γ_{mix}	Reference
		Cation1	Anion1	Cation2	Anion2		
	41	[C_4mim]	[BF_4]	[Bpy]	[BF_4]	1.00	[11]
	42	[C_6mim]	[BF_4]	[Bpy]	[BF_4]	1.01	[11]
	43	[C_4mim]	[TF_2N]	[P4444]	[TF_2N]	1.03	[14]
	44	[C_4mim]	[TF_2N]	[NMe_4]	[TF_2N]	1.05	[14]
	45	[C_4mim]	[BF_4]	[C_8mim]	[BF_4]	1.03	[7]
	46	[C_4mim]	[TF_2N]	[PPh_4]	[TF_2N]	1.05	[14]
	47	[C_6mim]	[TF_2N]	[C_2mim]	[TF_2N]	1.03	[11]
	48	[C_{10}mim]	[TF_2N]	[C_4mim]	[TF_2N]	1.03	[17]
	49	[C_8mim]	[TF_2N]	[C_4mim]	[TF_2N]	1.01	[17]
	50	[C_6mim]	[TF_2N]	[C_4mim]	[TF_2N]	1.00	[17]
	51	[C_5mim]	[TF_2N]	[C_4mim]	[TF_2N]	1.00	[17]
	52	[C_2mim]	[TF_2N]	[C_4mim]	[TF_2N]	1.01	[17]
	53	[C_1mim]	[TF_2N]	[C_4mim]	[TF_2N]	1.02	[17]
**Distinct Ions ILs (Miscible)**	54	[C_4mim]	[BF_4]	[C_8mim]	[TF_2N]	1.11	[18]
	55	[C_2mim]	[TF_2N]	[C_4mim]	[$EtSO_4$]	0.88	[4]
	56	[C_2mim]	[TF_2N]	[C_4mim]	[PF_6]	1.02	[11]
	57	[P66614]	[Ac]	[C_6mim]	[TF_2N]	0.25	[11]
	58	[P66614]	[3-Triz]	[C_4mim]	[TF_2N]	0.58	[12]
	59	[P66614]	[3-Triz]	[C_6mim]	[TF_2N]	0.54	[12]
	60	[P66614]	[4-Triz]	[C_6mim]	[TF_2N]	—	[12]
	61	[P66614]	[tzole]	[C_6mim]	[TF_2N]	0.90	[12]
**ILs Mixture (Immiscible)**	62	[C_1mim]	[Cl]	[P66614]	[Cl]	1.80	[19]
	63	[C_2mim]	[Cl]	[P66614]	[Cl]	1.63	[19]
	64	[C_3mim]	[Cl]	[P66614]	[Cl]	1.56	[19]
	65	[C_4mim]	[Cl]	[P66614]	[Cl]	1.50	[19]
	66	[C_5mim]	[Cl]	[P66614]	[Cl]	1.46	[19]
	67	[C_2mim]	[$MeSO_3$]	[P66614]	[TF_2N]	1.84	[19]
	68	[C_3mpyr]	[TF_2N]	[P66614]	[TF_2N]	1.45	[16]
	69	[C_2mim]	[TF_2N]	[P66614]	[TF_2N]	1.44	[20]
	70	[Prmpyr]	[TF_2N]	[P66614]	[TF_2N]	1.32	[20]
	71	[Epy]	[TF_2N]	[P66614]	[TF_2N]	1.46	[20]
	72	[EthAM]	[CHO_2]	[C_{10}mim]	[TF_2N]	8.22	[20]
	73	[HC_2mim]	[BF_4]	[C_{10}mim]	[TF_2N]	1.87	[20]
	74	[MeAM]	[NO_3]	[C_{10}mim]	[TF_2N]	8.05	[20]
	75	[C_2mim]	[HSO_4]	[C_{10}mim]	[TF_2N]	1.50	[20]
	76	[DMeAM]	[NO_3]	[C_{10}mim]	[TF_2N]	4.15	[20]
	77	[EthAM]	[CHO_2]	[C_8mim]	[PF_6]	5.58	[20]

S3 ML Models for Predicting IL–IL Miscibility

Two ML models have been developed in this study to efficiently predict the miscibility behavior of IL–IL mixtures. Computing activity coefficients using the COSMO-RS software is computationally intensive, particularly for large combinatorial spaces of ionic liquids. To address this challenge, a ReLU-activated artificial neural network (ReLU-ANN) regression model is constructed and trained on a large dataset of COSMO-RS–predicted activity coefficients. This model provides accurate and rapid estimates of activity coefficients across a broad range of IL–IL composition profiles. In addition to the regression model, a binary classification model is developed to distinguish between miscible and immiscible IL–IL pairs. This classifier enables fast screening of IL combinations without requiring any thermodynamic calculations.

The Loss–Epoch curve demonstrating the training performance of the regression model is presented in Figure S2, while the corresponding training curve for the binary classification model is shown in Figure S2. The model weights, bias parameters, and the complete source code for the miscibility prediction model are available at the following link: `https://github.com/chenahid/Ionic-Liquid-Miscibility/releases/tag/Miscibility`

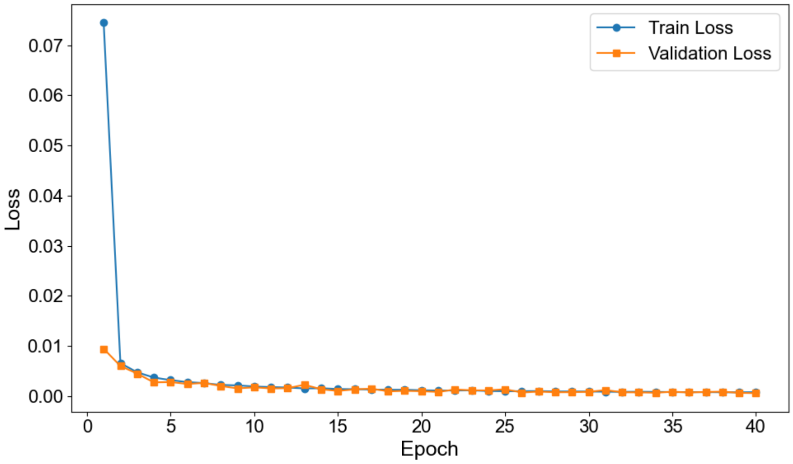

Figure S1: Training and validation loss curves over 40 epochs for the regression model.

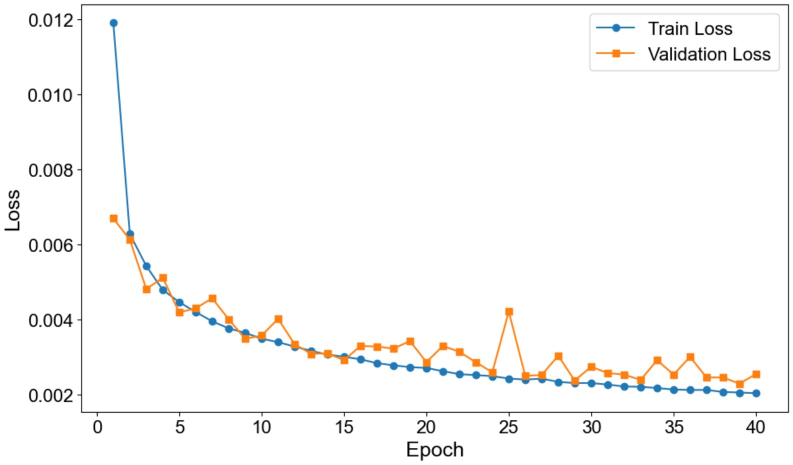

Figure S2: Training and validation loss curves over 40 epochs for the binary classification model.

S4 Optimized Process Conditions Across IL–IL Compositions

Table S4 summarizes the optimized operating conditions, product purities, and energy requirements obtained for different [C_2mim][TF_2N] compositions in [C_2mim][TF_2N]+[C_2mim][SCN] mixture. As the [C_2mim][TF_2N] fraction increases, both purities and total duty change non-monotonically, revealing a clear optimum near 0.57 wt/wt TF_2N where the system achieves maximum product purity with a moderate energy penalty.

Table S4: Optimized process variables, product purities, and total duty at different $[C_2mim][TF_2N]$ compositions in $[C_2mim][TF_2N]+[C_2mim][SCN]$ mixture.

TF_2N Comp (wt/wt)	Feed Stage –	Reflux Ratio –	IL Flow (kg/h)	Flash P (MPa)	Flash T (K)	IL Temp (K)	R125 Purity (wt/wt)	R32 Purity (wt/wt)	Total Duty (kJ/kg)
0.00	24	2.45	24	0.112	366.90	293.04	0.9228	0.9228	485.77
0.06	24	2.24	24	0.090	366.77	289.69	0.9301	0.9301	496.00
0.12	28	4.17	24	0.090	357.22	291.27	0.9392	0.9392	524.61
0.18	28	3.86	24	0.090	373.00	278.00	0.9473	0.9473	620.70
0.24	26	4.11	24	0.090	373.00	278.00	0.9535	0.9535	638.27
0.30	26	4.11	24	0.090	373.00	278.00	0.9579	0.9579	648.28
0.36	26	4.11	24	0.090	373.00	278.00	0.9617	0.9617	658.43
0.42	26	7.00	23.31	0.090	373.00	278.00	0.9642	0.9641	744.06
0.48	25	7.00	24	0.103	373.00	278.00	0.9675	0.9674	766.35
0.53	23	5.46	24	0.090	373.00	278.00	0.9699	0.9699	726.95
0.57	23	5.46	24	0.090	373.00	278.00	0.9707	0.9706	734.10
0.63	23	5.46	24	0.090	373.00	278.00	0.9707	0.9706	745.24
0.69	23	5.46	24	0.090	373.00	278.00	0.9688	0.9686	757.05
0.75	22	5.82	24	0.090	373.00	278.00	0.9643	0.9642	780.79
0.81	22	5.82	24	0.090	373.00	278.00	0.9556	0.9556	795.22
0.87	22	5.82	24	0.090	373.00	278.00	0.9412	0.9412	811.69
0.93	22	5.82	24	0.090	373.00	278.00	0.9193	0.9193	830.77
1.00	22	5.82	24	0.090	373.00	278.00	0.8766	0.8799	852.87

References

[1] Annat, G.; MacFarlane, D. R.; Forsyth, M. Transport properties in ionic liquids and ionic liquid mixtures: the challenges of NMR pulsed field gradient diffusion measurements. *The Journal of Physical Chemistry B* **2007**, *111*, 9018–9024.

[2] Santiago, A.; Mert, A. Mixed Ionic Liquids: The Case of Pyridinium-Based Fluids. *The Journal of Physical Chemistry B* **2012**, *116*, 25262537.

[3] Xiao, D.; Rajian, J. R.; Li, S.; Bartsch, R. A.; Quitevis, E. L. Additivity in the optical Kerr effect spectra of binary ionic liquid mixtures: implications for nanostructural organization. *The Journal of Physical Chemistry B* **2006**, *110*, 16174–16178.

[4] Pinto, A. M.; Rodríguez, H.; Colón, Y. J.; Arce Jr, A.; Arce, A.; Soto, A. Absorption of carbon dioxide in two binary mixtures of ionic liquids. *Industrial & Engineering Chemistry Research* **2013**, *52*, 5975–5984.

[5] Stoppa, A.; Buchner, R.; Hefter, G. How ideal are binary mixtures of room-temperature ionic liquids? *Journal of Molecular Liquids* **2010**, *153*, 46–51.

[6] Navia, P.; Troncoso, J.; Romaní, L. Excess magnitudes for ionic liquid binary mixtures with a common ion. *Journal of Chemical & Engineering Data* **2007**, *52*, 1369–1374.

[7] Potdar, S.; Anantharaj, R.; Banerjee, T. Aromatic extraction using mixed ionic liquids: experiments and COSMO-RS predictions. *Journal of Chemical & Engineering Data* **2012**, *57*, 1026–1035.

[8] García, S.; Larriba, M.; García, J.; Torrecilla, J. S.; Rodríguez, F. Liquid–liquid extraction of toluene from n-heptane using binary mixtures of N-butylpyridinium tetrafluoroborate and N-butylpyridinium bis (trifluoromethylsulfonyl) imide ionic liquids. *Chemical Engineering Journal* **2012**, *180*, 210–215.

[9] Vataščin, E.; Dohnal, V. Thermodynamic properties of aqueous solutions of [EMIM] thiocyanate and [EMIM] dicyanamide. *The Journal of Chemical Thermodynamics* **2017**, *106*, 262–275.

[10] Almeida, H. F.; Canongia Lopes, J. N.; Rebelo, L. P.; Coutinho, J. A.; Freire, M. G.; Marrucho, I. M. Densities and viscosities of mixtures of two ionic liquids containing a common cation. *Journal of Chemical & Engineering Data* **2016**, *61*, 2828–2843.

[11] Chen, Y.; Ma, S.; Lei, Y.; Liang, X.; Liu, X.; Kontogeorgis, G. M.; Gani, R. Ionic liquid binary mixtures: Machine learning-assisted modeling, solvent tailoring, process design, and optimization. *AIChE Journal* **2024**, *70*, e18392.

[12] Fillion, J. J.; Brennecke, J. F. Viscosity of ionic liquid–ionic liquid mixtures. *Journal of Chemical & Engineering Data* **2017**, *62*, 1884–1901.

[13] Lepre, L. F.; Costa Gomes, M.; Padua, A. A.; Ando, R. A.; Ribeiro, M. C. On the regular behavior of a binary mixture of ionic liquids. *The Journal of Physical Chemistry B* **2019**, *123*, 6579–6587.

[14] Weber, C. C.; Masters, A. F.; Maschmeyer, T. Controlling hydrolysis reaction rates with binary ionic liquid mixtures by tuning hydrogen-bonding interactions. *The Journal of Physical Chemistry B* **2012**, *116*, 1858–1864.

[15] Oliveira, M.; Dominguez-Perez, M.; Cabeza, O.; Lopes-da Silva, J.; Freire, M.; Coutinho, J. Surface tensions of binary mixtures of ionic liquids with bis (trifluoromethylsulfonyl) imide as the common anion. *The Journal of Chemical Thermodynamics* **2013**, *64*, 22–27.

[16] Annat, G.; Forsyth, M.; MacFarlane, D. R. Ionic Liquid Mixtures Variations in Physical Properties and Their Origins in Molecular Structure. *The Journal of Physical Chemistry B* **2012**, *116*, 8251–8258.

[17] Chen, Y.; Liu, X.; Woodley, J. M.; Kontogeorgis, G. M. Gas solubility in ionic liquids: UNIFAC-IL model extension. *Industrial & Engineering Chemistry Research* **2020**, *59*, 16805–16821.

[18] Lei, Z.; Han, J.; Zhang, B.; Li, Q.; Zhu, J.; Chen, B. Solubility of CO_2 in binary mixtures of room-temperature ionic liquids at high pressures. *Journal of Chemical & Engineering Data* **2012**, *57*, 2153–2159.

[19] Arce, A.; Earle, M. J.; Katdare, S. P.; Rodríguez, H.; Seddon, K. R. Mutually immiscible ionic liquids. *Chemical Communications* **2006**, 2548–2550.

[20] Omar, S.; Lemus, J.; Ruiz, E.; Ferro, V. R.; Ortega, J.; Palomar, J. Ionic Liquid Mixtures An Analysis of Their Mutual Miscibility. *The Journal of Physical Chemistry B* **2014**, *118*, 2442–2450.
